# Fell‐Muir Lecture: Fibrillin microfibrils: structural tensometers of elastic tissues?

**DOI:** 10.1111/iep.12239

**Published:** 2017-09-14

**Authors:** Cay M. Kielty

**Affiliations:** ^1^ Wellcome Trust Centre for Cell‐Matrix Research School of Biological Sciences Faculty of Biology, Medicine and Health University of Manchester Manchester UK

**Keywords:** elastic fibres, fibrillin, fibrillinopathies, microfibrils

## Abstract

Fibrillin microfibrils are indispensable structural elements of connective tissues in multicellular organisms from early metazoans to humans. They have an extensible periodic beaded organization, and support dynamic tissues such as ciliary zonules that suspend the lens. In tissues that express elastin, including blood vessels, skin and lungs, microfibrils support elastin deposition and shape the functional architecture of elastic fibres. The vital contribution of microfibrils to tissue form and function is underscored by the heritable fibrillinopathies, especially Marfan syndrome with severe elastic, ocular and skeletal tissue defects. Research since the early 1990s has advanced our knowledge of biology of microfibrils, yet understanding of their mechanical and homeostatic contributions to tissues remains far from complete. This review is a personal reflection on key insights, and puts forward the conceptual hypothesis that microfibrils are structural ‘tensometers’ that direct cells to monitor and respond to altered tissue mechanics.

## Introduction

This review integrates the knowledge of fibrillin microfibrils gained over the past 25 years with recent discoveries, and draws on this framework to deliver the latest insights into the pathobiology of the fibrillinopathies including Marfan syndrome. It recounts the discovery of microfibrils and their initial characterization, details their molecular composition, tissue‐specific functional architecture and cellular interactions, and evaluates microfibril structure and models of their molecular organization and extensibility. This comprehensive backdrop provides the context for a hypothetical model of how microfibrils may act as structural ‘tensometers’ to modulate TGF‐β‐driven tissue remodelling, by undergoing conformation changes upon extension that alter integrin receptor specificity and cellular responses.

## Microfibrils of the extracellular matrix

**Figure 1:**
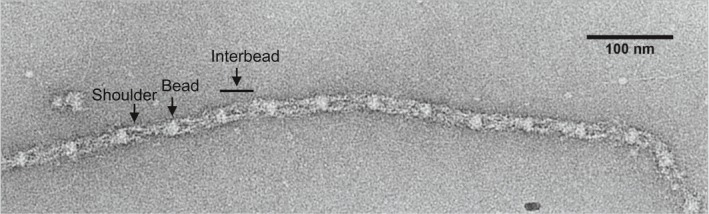
Electron microscopy image of a negatively stained tissue‐isolated microfibril. The image shows microfibril bead, shoulder and interbead features. Scale bar = 100 nm. Image provided by A. Godwin and C. Baldock.

Microfibrils are abundant components of the extracellular matrix of multicellular organisms from hydromedusae to humans (Cleary & Gibson 1983; Baldwin *et al*. 2013; Sengle & Sakai 2015). They are found in dynamic tissues that are defined as subject to constant stretch and recoil (e.g. lung, blood vessels, skin), and in deformable tissues such as perichondrium, sclera and cornea. They are linear beaded assemblies (Figure 1) that support tissue elasticity, and in mammals act as scaffold for elastin during elastic fibre formation. They also influence the bioavailability of TGF‐β growth factors that direct tissue morphogenesis and remodelling. Since the early 1990s, huge advances made in defining their assembly, organization and functions have shed light on their genotype–phenotype links in the fibrillinopathies.

## Discovery

The existence of bundles of fine filamentous microfibrils within dynamic connective tissues such as skin and aorta has been known for many years (Hall 1951; Low 1962; Ross & Bornstein 1969). Hall identified an elastase‐resistant glycoprotein in human and ox elastic tissue extracts, whilst Low observed the presence of thin microfibrillar arrays in elastic tissue matrices. Ross and Bornstein developed an extraction protocol based on collagenase digestion and reductive guanidine extractions. Microfibrils were seen to support elastin deposition and to associate with mature elastic fibres. Early studies revealed their anionic staining characteristics and that they comprise collagenase‐resistant glycoproteins. A major glycoprotein of apparent M_r_ 300,000, designated microfibrillar protein (MFP) II, was then identified (Sear *et al*. 1978; Sear *et al*. 1981a; Sear *et al*. 1981b). MFPII from microfibril‐rich foetal bovine nuchal ligament fibroblast cultures was metabolically labelled, immunoprecipitated with anti‐microfibrillar protein serum and analysed by SDS‐PAGE and gel filtration. Subsequently, a glycoprotein with similar features, named fibrillin, was isolated from human fibroblast cell cultures (Sakai *et al*. 1986). As MFPII and fibrillin‐1 had similar electrophoretic mobilities in reducing conditions and were both collagenase resistant (Sakai *et al*. 1986), it seems highly likely (but was never confirmed) that they were the same molecule. Microfibril bead and interbead elements were identified, and corresponding pepsin‐resistant fragments were isolated (Maddox *et al*. 1989). Keene *et al*. (1991) showed that dermal tissue microfibrils are periodic using immunogold electron microscopy. We developed protocols to isolate native microfibrils from tissues and cell layers, using collagenase and size fractionation, which enabled analysis of microfibril organization (Kielty *et al*. 1991). Also in 1991, the seminal discovery was made that mutations in the gene encoding fibrillin (now designated fibrillin‐1) on chromosome 15q21.1 cause Marfan syndrome, a severe disease affecting aorta, eyes, skin, lung and long bones (Dietz *et al*. 1991; Maslen *et al*. 1991). A related disease, congenital contractural arachnodactyly (Beals syndrome), with skeletal and ocular defects, was linked to mutations in the fibrillin‐2 gene on chromosome 5q23.3 (Lee *et al*. 1991).

These findings were the starting gun to resolve the nature of microfibrils, how they form and function in tissues, how they contribute to matrix mechanics and regulate bioavailability of TGF‐β growth factors and how mutations in fibrillin genes cause fibrillinopathies.

## Tissue‐specific architecture

In dynamic tissues of lower organisms, extensive parallel bundles of microfibrils intercalate with collagen fibres, as in sea cucumber dermis (Thurmond & Trotter 1996), and with cells as in lobster aortic medial layer where microfibril bundles are juxtaposed to smooth muscle cells (Faury 2001; Bussiere *et al*. 2006). Indeed, microfibrils are the primary structural matrix component of crustacean aortae, endowing elastic properties on these low pressure vascular systems (McConnell *et al*. 1996). In hydromedusae, reversible microfibril extension drives the refill stroke that deforms the mesogleal bell, thereby powering forward thrust (Reber‐Müller *et al*. 1995; Megill *et al*. 2005). Similar arrays of microfibrils occur in primitive vertebrates such as lamprey (Isokawa *et al*. 1989).

In mammals, microfibril architecture reflects its tissue‐specific location. In zonules, microfibril bundles are deposited by ciliary epithelial cells, and anchored at one end within the ciliary body muscle and the lens capsule at the other, thereby supporting lens dynamics. In elastin‐expressing tissues, elastic fibres form when elastin is deposited onto bundles of microfibrils (Wagenseil & Mecham 2007; Baldwin *et al*. 2013). The elastic network of skin comprises microfibril bundles (known as oxytalan fibres) that emanate from the dermal–epithelial junction into the dermis, where they intercalate with thin elastin‐containing fibres called elaunin fibres; these in turn form a continuum with thicker elastic fibres in the reticular dermis. In aorta, microfibrils are arranged circumferentially within the internal and external elastic laminae, and the medial elastic laminae that intercalate with smooth muscle cells; as structural components of vascular elastic fibres, microfibrils contribute to endowing elastic recoil to damp down pulsatile flow and pressure from the heart (Carta *et al*. 2009). In lung, microfibril‐associated elastin forms networks that allow alveolar expansion and retraction for breathing (Shifren *et al*. 2007). In auricular cartilage, elastic fibres form fine networks that endow flexible properties (Ito *et al*. 2001).

## Microfibril composition

Microfibrils are based on fibrillin and interact with other glycoproteins and proteoglycans.

## The fibrillin superfamily

**Figure 2:**
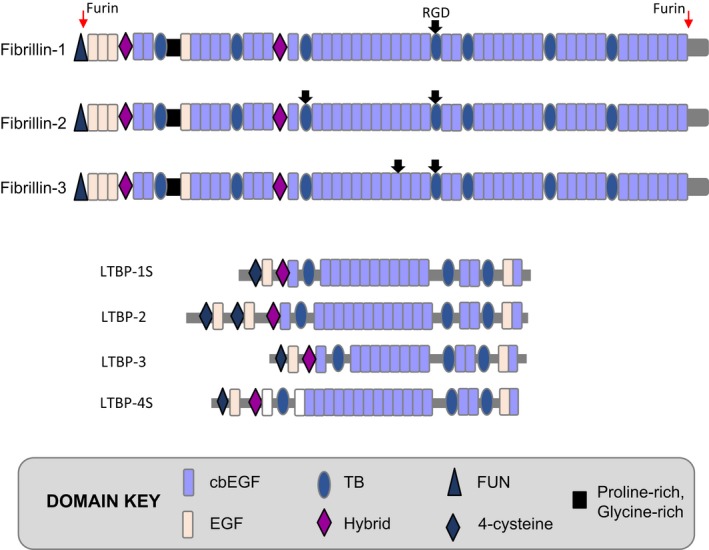
Fibrillin superfamily domain structures. [Colour figure can be viewed at wileyonlinelibrary.com].

The fibrillin superfamily has evolved to comprise three fibrillins and four latent TGF‐β binding proteins (LTBPs) (Figure 2).

### Fibrillins

Early studies of microfibrils (see ‘Discovery’) had identified fibrillin as the principal structural component (Sakai *et al*. 1986; Maddox *et al*. 1989). Genome analysis revealed that there are, in fact, three human fibrillin genes (Lee *et al*. 1991; Corson *et al*. 2004). This discovery, with *in situ* hybridization and immunomicroscopy, led to details of their tissue distribution and abundance (Quondamatteo *et al*. 2002; Sabatier *et al*. 2011; Hubmacher *et al*. 2014a). Fibrillin‐1 is by far the most abundant isoform through life and is the main form in adult tissues. Fibrillins‐2 and ‐3 are mainly expressed in developing tissues, and fibrillin‐2 is upregulated in healing wounds (Brinckmann *et al*. 2010). The fibrillin‐3 gene is disrupted in mice (Corson *et al*. 2004), so is not essential for all mammalian life. This review focuses mainly on fibrillin‐1 as it is by far the major isoform in terms of abundance and distribution, and its biology is much better defined that that of fibrillins‐2 and ‐3; however, comments are included below on these latter isoforms where data are available.

Sequence analysis revealed that fibrillin‐1 is a large glycoprotein of 2871 amino acids (Figure 2). It has a multidomain construction that is dominated by 43 calcium‐binding epidermal growth factor‐like (cbEGF) domains. Non‐calcium‐binding EGFs, TB domains (TGF‐β‐binding‐like domain, homologous to a TGF‐β‐binding domain in latent TGF‐β‐binding protein [LTBP)‐1]) and hybrid domains (with features of cbEGFs and TB domains) are also present. Notably, TB domains are a unique hallmark of the fibrillin superfamily. The unique N‐ and C‐terminal regions each contain a furin cleavage site, and the molecule has one Arg‐Gly‐Asp (RGD) cell adhesion motif and 14 predicted N‐glycosylation sites. Additionally, serine 2702 can be phosphorylated by the kinase FAM20C (Tagliabracci *et al*. 2015).

Fibrillins‐2 and ‐3 are highly homologous to fibrillin‐1, differing mainly in that the fibrillin‐1 proline‐rich region is replaced by glycine‐rich and glycine‐/proline‐rich regions respectively. Fibrillin‐3 also lacks the second cbEGF‐like domain. Microfibrils comprising fibrillin‐1 and fibrillin‐2 can co‐assemble in developing tissues (Charbonneau *et al*. 2003), and fibrillin‐3 also localizes to microfibrils in foetal tissues (Corson *et al*. 2004). Murine ciliary zonules contain fibrillins‐1 and ‐2, whereas both juvenile and adult human zonules are based primarily on fibrillin‐1 (Beene *et al*. 2013; Hubmacher *et al*. 2014a), highlighting species‐specific differences in fibrillin isoform distribution. This result confirmed our earlier proteomic analysis of microfibrils purified from zonules, aorta and skin, which had detected only the fibrillin‐1 isoform in human zonules, alongside low levels of the microfibril‐associated glycoprotein MAGP‐1 (see ‘Microfibril‐associated molecules’) (Cain *et al*. 2006). A recent proteomic analysis (De Maria *et al*. 2017) confirmed that fibrillin‐1 and MAGP‐1, with LTBP‐2, are the most abundant components of human zonules.

### LTBPs

The LTBPs are a family of four gene products, LTBPs 1–4 (Figure 2) (Todorovic & Rifkin 2012; Robertson *et al*. 2015). Whilst much smaller molecules than the fibrillins, they have many structural similarities, notably contiguous arrays of cbEGFs interspersed with TB domains. LTBPs 1 and 4 occur as long or short forms (Figure 2), and LTBPs 1, 3 and 4 can bind latent TGF‐β and are secreted from cells as large latent complexes (Todorovic & Rifkin 2012). These complexes can store latent TGF‐β in matrix, binding molecules such as fibronectin, fibrillin‐1 and heparan sulphate, until altered tissue mechanics trigger its release by αvβ integrin‐mediated cellular forces (Wipff *et al*. 2007; Massam‐Wu *et al*. 2010; Horiguchi *et al*. 2012; Sarrazy *et al*. 2014; Hinz 2015). Although LTBPs do not form beaded microfibrils, we have shown that they can assemble into branching filamentous arrays that are stabilized by transglutaminase‐2 and that this multimerization process is enhanced by heparan sulphate (Troilo *et al*. 2016).

### Evolution of the fibrillin superfamily

Evolutionary studies revealed that fibrillin molecules and microfibrils are among the most ancient and widespread structural matrix elements of multicellular organisms (see Tissue‐specific architecture) (Robertson *et al*. 2011; Piha‐Gossack *et al*. 2012; Baldwin *et al*. 2013). By phylogenetic analysis and multiple sequence alignments, we mapped the evolution of vertebrate fibrillins 1–3, invertebrate fibrillin and ancestral fibrillin‐like EGF array‐containing proteins (Baldwin *et al*. 2013). Invertebrate fibrillin and beaded microfibrils (see ‘Tissue‐specific architectures’) were present in many metazoan species 500–700 million years before the appearance of elastin ~300 million years ago in teleost fish, and the cell adhesion molecule fibronectin. The appearance within fibrillin of the integrin adhesion motif Arg‐Gly‐Asp (RGD), which as a motif first appears when vertebrates diverged from other chordates, highlights an emerging need for direct microfibril–cell communication along with robust mesenchymal tissues such as elastic arteries. LTBPs emerged ~300 million years after invertebrate fibrillins.

## Microfibril‐associated molecules

Many molecules can bind fibrillins, and some may endow tissue‐specific functions on microfibrils; for details of these microfibril‐associated molecules, see table 1 in Baldwin *et al*. (2013) and Zeyer and Reinhardt (2015). Several microfibril‐associated glycoproteins (M_r_s 25,000 to 340,000) were initially identified, including microfibril‐associated glycoprotein‐1 (MAGP‐1; MFAP2), LTBP‐2 (Gibson *et al*. 1989) and fibulins (El‐Hallous *et al*. 2007). Other microfibril‐associated molecules include MAGP‐2 (Hanssen *et al*. 2004; Mecham & Gibson 2015), fibronectin (Kinsey *et al*. 2008; Sabatier *et al*. 2009), chondroitin sulphate proteoglycans versican and decorin (Kielty *et al*. 1996; Trask *et al*. 2000; Isogai *et al*. 2002; Reinboth *et al*. 2002), the glycosaminoglycan hyaluronan (Murasawa *et al*. 2013) and heparan sulphate (HS) and the HS proteoglycans perlecan and syndecan‐4 (Tiedemann *et al*. 2001, 2005; Cain *et al*. 2005, 2008, 2016).

In their proposed role as regulators of the bioavailability of TGF‐β growth factors (see ‘Tall fibrillinopathies’), fibrillin‐1 can interact with LTBP‐1, which in turn binds latent TGF‐β (Todorovic & Rifkin 2012). However, latent TGF‐β also binds other matrix components such as fibronectin (Horiguchi *et al*. 2012), whilst LTBP‐1 also independently multimerizes in a heparin‐/HS‐dependent manner (Troilo *et al*. 2016). In contrast, fibrillin‐1 can directly bind prodomains of BMPs 2, 4, 5, 7 and 10, inducing a conformational change that blocks BMP interactions with its receptors (Gregory *et al*. 2005; Sengle *et al*. 2011; Wohl *et al*. 2016).

As the scaffold for elastin (see ‘Discovery’), microfibrils support the hierarchical elastic fibre assembly process (Wagenseil & Mecham 2007). They interact with tropoelastin, the small fibulins‐4 and ‐5, and lysyl oxidase (Rock *et al*. 2004; Choudhury *et al*. 2009; Baldwin *et al*. 2013). Using a recombinant approach, we identified an elastin–fibrillin‐1 transglutaminase link that may stabilize forming elastic fibres (Rock *et al*. 2004). ADAMTS10 can interact with fibrillin‐1 (Kutz *et al*. 2011; Hubmacher & Apte, 2015; Cain *et al*. 2016), and it colocalizes with microfibrils in the papillary dermis (Kutz *et al*. 2011). Its close homologue ADAMTS6 (Cain *et al*. 2016) and related ADAMTSL4 (Gabriel *et al*. 2012) and ADAMTSL6 (Tsutsui *et al*. 2010) also bind microfibrils. Moreover, ADAMTS17 binds fibrillin‐2, although not fibrillin‐1 (Hubmacher *et al*. 2017). Thus, these members of the ADAMTS(L) family are probably microfibril‐associated molecules *in vivo*.

## Fibrillinopathies and associated defects in tissue mechanics

**Figure 3:**
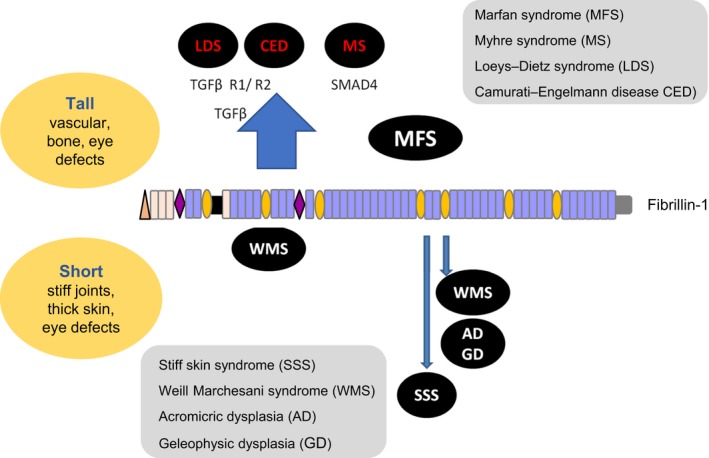
‘Tall’ and ‘short’ fibrillinopathies. [Colour figure can be viewed at wileyonlinelibrary.com].

The wide evolutionary distribution of fibrillin underscores the importance of microfibrils to the integrity and mechanical properties of tissues. Hence, it is not surprising that severe fibrillinopathies are caused by mutations in the fibrillin genes (Figure 3). Moreover, microfibrils are susceptible to degradation by serine proteases and metalloproteinases (Kielty *et al*. 1994; Ashworth *et al*. 1999c) and their degeneration through life appears to contribute to dermal ageing and to loss of elastic fibre integrity associated with emphysema and vascular ageing (Watson *et al*. 1999; Koenders *et al*. 2009; Mariko *et al*. 2011; Baldwin *et al*. 2013).

## ‘Tall’ fibrillinopathies

Most prominent of the heritable fibrillinopathies is autosomal dominant Marfan syndrome (MFS; MIM 154700), which is caused by mutations in the fibrillin‐1 gene on chromosome 15q21.1 (Ramirez & Dietz 2007). MFS causes life‐threatening aortic defects, and skeletal and ocular symptoms including scoliosis and ectopia lentis. Marfan‐related diseases also caused by mutations in fibrillin‐1 include ectopia lentis syndrome, familial thoracic aortic aneurysm and dissection, and MASS phenotype (mitral valve, myopia, aorta, skin and skeletal features). MFS has a frequency of ~1:5000, and numerous mutations, both familial and spontaneous, have been identified throughout fibrillin‐1 (http://www.umd.be/FBN1/). Most are missense mutations that disrupt cbEGF and TB domains. Other mutations include small insertions or deletions, which can result in premature termination codons, splice site or frameshift mutations with mRNA decay, and larger rearrangements. Interestingly, no mutations have been identified in the proline‐rich region, which might suggest that this region is indispensible to fibrillin biology (see ‘Models of fibrillin‐1 alignment in extensible microfibrils’).

Genotype–phenotype correlations in MFS are complex. Recurring examples are where missense mutations in cbEGF or TB domains perturb calcium binding and domain–domain interfaces, and/or expose cryptic proteolytically sensitive sites (Raghunath *et al*. 1995; McGettrick *et al*. 2000; Mellody *et al*. 2006); outcomes are structural changes and/or degradation. Some of the mutations at the start of a central block of 12 cbEGFs cause severe neonatal MFS; these mutations may disrupt fibrillin‐1 secretion (Whiteman & Handford 2003) and/or increase proteolytic sensitivity (Kirschner *et al*. 2011). Some mutations that reduce fibrillin‐1 production, such that microfibrils are ‘normal’ but reduced in number, cause less severe phenotypes than those where mutant fibrillin‐1 disrupts microfibrils. Certain mutations beyond the C‐terminal furin cleavage site cause MFS (see ‘Furin processing’). Others in C‐terminal exon 64 cause a marfanoid–progeroid–lipodystrophy syndrome (Passarge *et al*. 2016) by an unknown mechanism. Mutations that perturb furin cleavage of the C‐terminal propeptide of fibrillin‐1, which is a glucogenic protein hormone called asprosin (Romere *et al*. 2016), may alter glucose metabolism.

Murine models of MFS have focussed more on fibrillin‐1 mutation‐induced TGF‐β perturbations than on structural microfibril defects as disease ‘drivers’ (Sengle & Sakai 2015; Smaldone & Ramirez 2016). The hypomorphic mgΔ mouse had pathologically elevated TGF‐β activity in the lung (Neptune *et al*. 2003), which correlated with initial predictions that perturbations in large latent TGF‐β complexes interacting directly with fibrillin‐1 (see LTBPs) would manifest as enhanced TGF‐β signalling in affected tissues. Moreover, mutations in the TGF‐β receptors or signalling pathway cause severe diseases such as Loeys–Dietz syndrome, which are highly reminiscent of MFS (Lindsay *et al*. 2012). However, mice that lack the main LTBP‐1 binding domain on fibrillin‐1 (the first hybrid domain) assembled microfibrils and were healthy with no vascular disease (Charbonneau *et al*. 2010); thus, LTBP‐1 binding directly to microfibrils is unlikely directly to cause TGF‐β perturbations in MFS. Furthermore, genetic disruption of the TGF‐β receptor II in heterozygous C1039G mice did not prevent vascular damage (Lindsay *et al*. 2012). Another mouse model, C1039G, which recapitulates vascular features of MFS, also showed abnormal activation of TGF‐β. It was exploited to test potential therapies such as losartan, which is an angiotensin II type I receptor blocker that may also inhibit TGF‐β signalling; human losartan trials have shown variable therapeutic benefits (Lacro *et al*. 2014). Importantly, a biphasic response to TGF‐β neutralization in MFS mice was identified (Cook *et al*. 2015). It suggested that overactivation of the angiotensin II type I receptor and ‘protective’ TGF‐β signalling were initial vessel repair responses to mechanical changes, whereas arterial degeneration reflected pathological TGF‐β outcomes.

Overall, it seems likely that altered TGF‐β activity in MFS is an aggressive cellular repair response to a defective mechanical environment. Some fibrillin‐1 mutations may also disrupt BMP prodomain interactions with fibrillin‐1 (Wohl *et al*. 2016), altering BMP signals which regulate musculoskeletal growth.

## ‘Short’ fibrillinopathies

Given that MFS and related diseases predispose patients to vascular defects, bone overgrowth and hypomuscularity, it was surprising to discover that another group of genetic fibrillin‐1 diseases exists that has essentially the ‘opposite’ phenotypic spectrum. Such patients have symptoms such as short stature, thick fibrotic skin, limited vascular involvement and hypermuscularity (Le Goff & Cormier‐Daire 2012). These diseases include stiff skin syndrome (SSS, MIM 184900), which is caused by mutations in the RGD‐containing TB4 module, and manifests as diffuse skin fibrosis with abundant, abnormally arranged microfibrils and characteristic truncation of the oxytalan fibres emanating from the dermal–epidermal junction. Weill–Marchesani syndrome (WMS; MIM #277600) and the acromelic dysplasias (acromicric dysplasia [AD], MIM 102370; geleophysic dysplasia [GD], MIM 231050), caused by mutations in TB5, also have short stature, thick skin with abundant microfibrils, joint stiffness and ocular defects. One WMS‐causing N‐terminal three‐domain deletion mutation has been reported (Sengle *et al*. 2012). Apart from this exception, all mutations causing ‘short’ fibrillinopathies perturb the fibrillin‐1 cell adhesion region (see ‘Interactions with cell surface receptors’), so fibrillin‐1 ligation to cells is needed for tissue formation and homeostasis. The N‐terminal WMS mutation deletes a binding site for ADAMTS‐like molecules, which may contribute to this pathology.

## Microfibril organization and extensibility

Structural and recombinant biology approaches have revealed insights into the molecular basis of the extensible beaded organization of microfibrils.

## Molecular dimensions and microfibril periodicity

Fibrillin molecules purified from fibroblast culture medium were shown to have a length of 148 nm, diameter of 2.2 nm and to be flexible (Sakai *et al*. 1991). Recombinant full‐length fibrillin‐1 had similar dimensions, with some bends (Lin *et al*. 2002). Given these molecular dimensions, it was surprising that isolated native microfibrils were found to have a periodic repeat of 56 nm (Sherratt *et al*. 2001), whilst tissue microfibrils had periodicities of 50–56 nm (Keene *et al*. 1991; Wess *et al*. 1998a; Davis *et al*. 2002). The question was: ‘How do fibrillin molecules assemble into periodic microfibrils?’

## Calcium dependence

We and others had shown that the 56‐nm periodicity of isolated microfibrils depends upon bound calcium (Kielty & Shuttleworth 1993; Cardy & Handford 1998; Wess *et al*. 1998b; Wang *et al*. 2009). In the presence of chelators such as EGTA, periodicity often reduced to <45 nm, with diameter correspondingly increased. Low‐angle X‐ray diffraction of intact hydrated zonules revealed that calcium chelation also altered tissue periodic structure (Wess *et al*. 1998b).

Structural dependence on calcium was explained at a molecular level by a series of high‐resolution NMR and crystallography studies (Handford 2000; Jensen *et al*. 2005). These studies showed, for bacterially expressed and refolded domain pairs and short arrays, that calcium binding by contiguous cbEGF‐like domains induces an extended arrangement. Many missense MFS mutations disrupt calcium binding by cbEGFs (Whiteman *et al*. 2001; Smallridge *et al*. 2003), underlining their essential importance to microfibril integrity. These studies showed how calcium regulates the molecular shape of fibrillin‐1.

## Exposed fibrillin‐1 sites

Many clues exist to the regions of assembled fibrillin‐1 that are exposed or cryptic.

### Cross‐link sites

**Figure 4:**
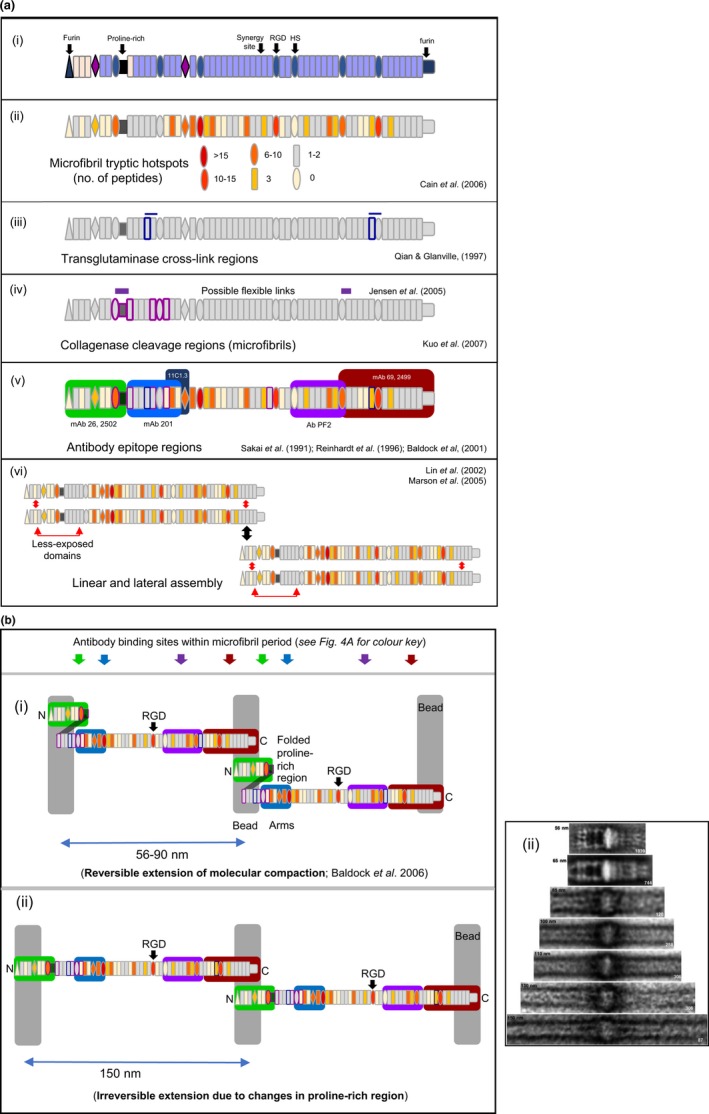
(a) Fibrillin‐1 features within assembled microfibrils. (i) Domain structure, showing N‐ and C‐terminal furin cleavage sites, the proline‐rich region and the central cell adhesion region. (ii) Hotspots for tryptic digestion of native microfibrils (Cain *et al*. 2006). (iii) Sites within assembled microfibrils adjacent to transglutaminase cross‐links (blue) (Qian & Glanville 1997). (iv) Sites that can be cleaved by bacterial collagenase (Kuo *et al*. 2007), and a possible flexible link between TB6 and following cbEGF domain (Jensen *et al*. 2005). (v) Boxes show the domains within which lie the epitopes to antifibrillin‐1 antibodies (Sakai *et al*. 1991; Reinhardt *et al*. 1996; Baldock *et al*. 2001). (vi) N‐to‐C‐, N‐to‐N‐ and C‐to‐C‐terminal interactions, which probably support linear and lateral assemblies (Lin *et al*. 2002; Marson *et al*. 2005). (b) Unstaggered fibrillin‐1 alignment model. (i) This model predicts folding at the proline‐rich region stabilized by N‐terminal interactions (see Figure 4a vi) (Marson *et al*. 2005), and reversible and irreversible extension. (ii) A comparison with observed extensions is shown (Wang *et al*. 2009). This image is reproduced with permission from J. Mol. Biol. (Elsevier). [Colour figure can be viewed at wileyonlinelibrary.com].

Transglutaminase cross‐links, involving sites towards N‐ and C‐terminal ends of fibrillin‐1, were discovered in microfibrils (Qian & Glanville 1997) (Figure 4a). They provided a clue to fibrillin‐1 alignment within microfibrils, although they may form between adjacent microfibrils. Either way, the cross‐link sequences are probably surface‐accessible and necessary to stabilize these extensible polymers.

### Proteolytic sites

Proteomic analysis of microfibrils from zonules, aorta and skin had revealed tryptic hotspots on microfibrillar fibrillin‐1 (Figure 4a), including a region within the N‐terminal half and the more C‐terminal RGD motif, but not the N‐ and C‐termini themselves (Cain *et al*. 2006). They suggest surface accessibility within native microfibrils. Two fibrillin‐1 sites cleaved by crude bacterial collagenase (at/near the proline‐rich region and TB2) are also accessible in microfibrils (Kuo *et al*. 2007).

### Epitope sites

Antibody epitope mapping localized several fibrillin‐1 sequences within microfibrils (Figure 4a). Sakai *et al*. (1991) had shown that monoclonal antibodies (mAbs) 201 (epitope within residues 451–909) and 69 (epitope within residues 2093–2871) gave a double banding pattern consistent with a parallel head‐to‐tail linear assembly. The epitopes for mAbs 26 (epitope within residues 45–450) and mAb 69 were near N‐ and C‐termini, and bound microfibrils either side of the bead, whilst mAb 201 bound to one side of the interbead (Reinhardt *et al*. 1996). We mapped the epitope for an antibody to pepsin‐resistant fragment PF2 (Maddox *et al*. 1989), which falls within the region comprising TB5, cbEGFs 25–31 and TB6 (Glanville *et al*. 1994) (Figure 4a), to the microfibril interbead (Baldock *et al*. 2001), and the epitope for mAb 11C1.3 (within N‐terminal residues 723–909; cbEGFs 7–9 and second hybrid domain) to one side of the bead. We also showed that mAbs 2502 and 2499 (also known as 26 and 69) bind either side of the bead. MAGP‐1, which bound to one side of the beads (Henderson *et al*. 1996), interacts with N‐terminal fibrillin‐1 (Mecham & Gibson 2015).

In summary, microfibrils comprise contiguous arrays of eight parallel molecules with N‐ and C‐termini proximal to the bead. The interbead is based on sequences from N‐ and C‐terminal halves of fibrillin‐1 (see ‘Models of fibrillin‐1 alignment in extensible microfibrils’). Much of the N‐terminal half and some of the C‐terminal half are exposed in native microfibrils.

## Cross‐section

Early electron microscopy studies had reported that tissue microfibrils had a translucent core and six to eight molecules in cross‐section (Cleary & Gibson 1983). Extracted beads were seen to have up to eight arms emanating from the bead structure (Keene *et al*. 1991). Baldock *et al*. (2001) predicted eight molecules in cross‐section from automated electron tomography and STEM mass mapping. In 2008, Hubmacher and co‐workers showed that C‐terminal multimerization induced bead structures with 8–12 arms (see ‘Homotypic interactions’). Single particle averaging of tissue microfibrils further delineated the lateral packing of microfibrils, confirming eight molecules arranged around a ‘hollow’ core (Wang *et al*. 2009). This arrangement implies tight spatial regulation of lateral interactions.

## Microfibril features in untensioned and extended states

Major structural features give clues to how fibrillin‐1 molecules assemble into microfibrils that, through changes in fibrillin‐1 organization, can extend and retract in dynamic tissues.

### Topography

Microfibrils extracted from tissues using denaturing guanidinium chloride were extensive beaded polymers with loosely organized interbead filaments (Keene *et al*. 1991), implying stabilization by covalent cross‐links. Extensive native microfibrils isolated from tissues and cell cultures with bacterial collagenase and size fractionation (Kielty *et al*. 1991) enabled analysis by rotary shadowing, scanning transmission electron microscopy (STEM) mass mapping, negative staining (Figure 1), atomic force microscopy (Kielty *et al*. 2002) and cryo‐negative staining (hydrated microfibrils without surface influences) (Wang *et al*. 2009). In all these approaches, microfibrils had an asymmetric ‘shoulder’ feature, confirming directionality. The asymmetric shoulder feature could represent folding within fibrillin‐1 although, because most of fibrillin‐1 comprises contiguous arrays of cbEGF‐like domains that have extended (although not fully linear) characteristics, sites capable of such folding may be limited to the proline‐rich region, the termini and the TB6–cbEGF32 interface (Jensen *et al*. 2005).

Automated electron tomography resolved the microfibril repeat period to 18.6 Å, with STEM mass mapping and mapping of antibody epitopes (see ‘Epitope sites’), and suggested that flexible fibrillin‐1 molecules span a single period in untensioned state (Baldock *et al*. 2001). Such packing was proposed to reflect flexibility at the proline‐rich region, the N‐ and C‐termini and at the TB5–cbEGF domain interface. Subsequently, using long recombinant fragments expressed in mammalian cells to ensure disulphide bonding and N‐glycosylation, we showed that calcium‐bound cbEGF‐like domain arrays in solution are not linear, as had been proposed from crystallography and NMR (see ‘Calcium dependence’), but are in fact somewhat compacted (Baldock *et al*. 2006). This feature must contribute to compaction of full‐length fibrillin‐1 within a microfibril period. Examination of microfibrils isolated with highly purified collagenase, or mechanically (no collagenase), enabled single particle averaging, which revealed further details of microfibrils including interbead, shoulder, bead and arms with lateral striations, and eight‐fold symmetry (Lu *et al*. 2006).

### Extension

As structural elements of dynamic connective tissues, microfibrils contribute to elasticity both as microfibril bundles and as components of mammalian elastic fibres. Biomechanical forces on invertebrate microfibril‐based tissues alter microfibril orientation and periodicity (see ‘Tissue‐specific architecture’). In mammalian dermis, immunomicroscopy showed that periodicity of tissue microfibrils can be extended to 76–80 nm and that ‘minimally processed’ microfibrils can be extended to 82–110 nm (Keene *et al*. 1991). X‐ray diffraction revealed that whole hydrated bovine zonules were reversibly extensible within the periodic range 56–80 nm (Wess *et al*. 1998a). Stretched whole zonular filaments showed reversible microfibril diffraction spacings of 60–70 nm and 90–100 nm with corresponding decreases in microfibril diameter, and also 160‐nm periodicity (Glab & Wess 2008). Together, these data indicate that tissue microfibrils are reversibly extensible, at least between periodicities of 56 nm and ~90 nm. As such, they are unique structural elements of multicellular evolution with inherent pliancy that endow tissues with extensile properties and a platform for cells to sense tissue mechanics (see ‘Microfibrils as structural tensometers?’).

Individual microfibrils with periodicities greater than 56 nm have been reported (Keene *et al*. 1991; Reinhardt *et al*. 1996; Kielty *et al*. 2002). By molecular combing, we demonstrated that isolated microfibrils could be extended to periodicities of ~80 nm and that they may act as stiff reinforcing filaments (Sherratt *et al*. 2003). Similarly, surface tension extended microfibrils to ~90 nm (Baldock *et al*. 2001). Kuo *et al*. (2007) proposed that cleavage by crude bacterial collagenase could account for observed extension of isolated microfibrils.

Baldock and co‐workers, using highly purified bacterial collagenase or no collagenase at all, found that microfibril periodicity was extended from 56 to 154 nm simply by decreasing ionic strength in the presence of calcium (Wang *et al*. 2009). These extensions were accounted for by gross interbead changes, with four fine filaments at periodicities less than 85 nm becoming two long thick intact filaments of ~150 nm (effectively the length of a fibrillin‐1 molecule) at higher periodicities (Figure 4b). Thus, the 56‐nm untensioned periodicity is stabilized by ionic interactions, which may drive retraction. Assuming eight molecules in cross‐section (see ‘Structural features’), the four filaments seen in untensioned 56‐nm periodic microfibrils might be four groups of two molecules, whilst the two long thick filaments seen at periodicities >150 nm might be two groups of four molecules.

Overall, these data suggest that fibrillin‐1 molecules are both folded and compacted (with asymmetric shoulder; *see above*) in untensioned microfibrils. Reversible extension to 90 nm could be achieved by straightening or compaction of the cbEGF‐like domain arrays driven by ionic interactions (Wang *et al*. 2009), whilst (possibly irreversible) extension beyond 90 nm could involve unfolding of the shoulder feature.

## Models of fibrillin‐1 alignment in extensible microfibrils

Several models of fibrillin alignment in microfibrils have been proposed. The first model, a linear head‐to‐tail arrangement, was based on the double banding pattern of mAbs 201 and 69, which recognized epitopes at N‐ and C‐termini of fibrillin‐1 respectively (Sakai *et al*. 1991). Recombinant full‐length fibrillin‐1 was also linear, with flexibility, and formed non‐overlapping dimers and trimers, implying only N‐ to C‐terminal interactions (Lin *et al*. 2002). Sakai and co‐workers proposed an unstaggered model to account for observed microfibril periodic extension, whilst immunolocalization of mAb epitopes (see ‘Exposed fibrillin‐1 sites’) had suggested that fibrillin‐1 is ‘compacted’ within tissue microfibrils (Reinhardt *et al*. 1996). We proposed a similar unstaggered model on the basis of structural imaging, extensibility, mAb epitopes and STEM mass mapping (Baldock *et al*. 2001; Wang *et al*. 2009) (see ‘Microfibril features’). Initially, we suggested several molecular folding events to account for compaction within a single period; subsequently, Baldock *et al*. (2006) showed that fibrillin‐1 molecules in solution are partly compacted. An updated version of this model, with folding at the proline‐rich region and observed molecular compaction, is presented (Figure 4b). This unstaggered model suggests that fibrillin‐1 molecules are compacted within a bead period and can extend and retract reversibly within the range 56–90 nm. It is consistent with the ability to induce microfibril extension by reducing ionic strength (Wang *et al*. 2009) (see ‘Extension’). However, the structure of the fibrillin‐1 proline‐rich region and its contribution to molecular flexibility are essentially unknown, with no homologous structures in the Protein Data Bank (Baldock *et al*. 2006). One‐dimensional NMR did show that it was unstructured and lacked secondary structures, and predicted that it has a random coil structure (Ashworth *et al*. 1999b). It is unknown whether the glycine‐rich and glycine‐/proline‐rich sequences of fibrillins‐2 and ‐3 are similarly unstructured.

Sakai and colleagues then proposed a 50% staggered model, with the inner C‐terminal half overlapped and internal to the outer N‐terminal half of the following molecule (Kuo *et al*. 2007). This model also fits the mapped antibody epitope sites. Given that fibrillin‐1 molecules are ~150 nm in length and that an untensioned bead period is 56 nm, this model implies that each half of fibrillin‐1 is compacted from ~75 to 56 nm in resting state, which may be the periodic extension range predicted by this model. It is unclear how it can account for the observed shoulder and ‘arm’ features of untensioned microfibrils, or the ~150‐nm extended periodicity of large microfibril polymers (physiological or not). Furthermore, the RGD and antibody epitope site for fragment PF2 in the C‐terminal half appear to be exposed on untensioned microfibrils (see ‘Exposed fibrillin‐1 sites’).

A third model with a 1/3 stagger was based on extrapolation from high‐resolution structural analysis of calcium‐bound short fibrillin‐1 domain pairs and short fragments, which adopted an extended linear organization (Handford 2000). However, fibrillin‐1 multidomain arrays in calcium solution are in fact somewhat compacted (Baldock *et al*. 2006).

It remains uncertain precisely how fibrillin‐1 aligns within microfibrils. In fact, unstaggered and staggered models can both accommodate all key alignment points and microfibrillar features, depending on exact packing and compaction predictions. We need definitive data on whether ionic interactions (see ‘Microfibril features: Extension’) do drive microfibril ‘retraction’ between 56 and 90 nm, and whether irreversible extension to ~150 nm involves unfolding of the proline‐rich region. Final resolution may await experimental cross‐linking to define multiple fibrillin‐1 alignment sites.

## Assembly of a microfibril

**Figure 5:**
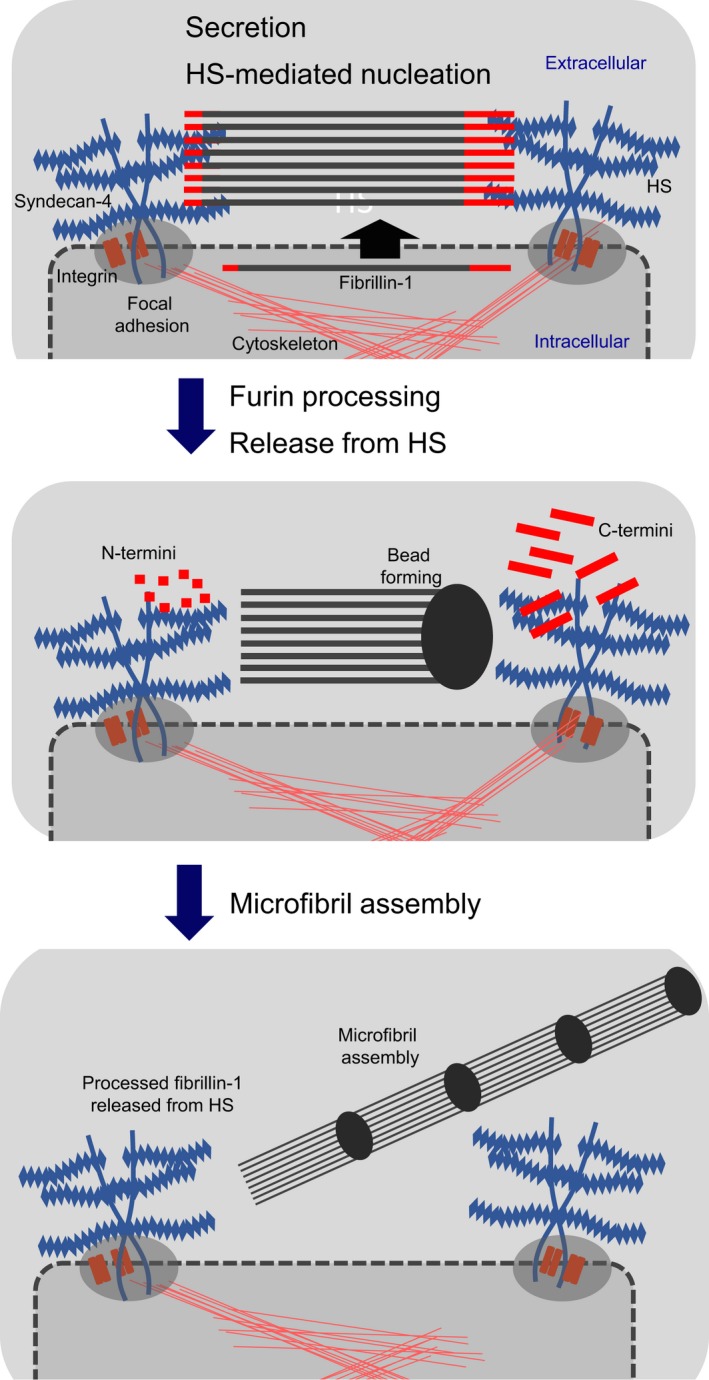
Model of fibrillin‐1 cell surface assembly. Unprocessed fibrillin‐1 is secreted, nucleated at the cell surface by focal adhesion HS interactions with N‐ and C‐termini, processed by furin and released from HS, enabling assembly into microfibrils. [Colour figure can be viewed at wileyonlinelibrary.com].

Much is now known about how newly secreted fibrillin‐1 molecules interact to form linear microfibrils (Figure 5). Outstanding issues include: ‘Is assembly of fibrillin‐1 part‐directed by cells?’ ‘How does HS influence assembly?’

## Furin processing

Fibrillin‐1 has N‐terminal (RAKRR) and C‐terminal (RKRR) furin cleavage sites. Milewicz and colleagues showed that fibrillin‐1 undergoes C‐terminal furin processing (removal of ~20 kDa) upon secretion (Raghunath *et al*. 1999); by SDS‐PAGE, it is difficult to detect N‐terminal processing (removal of 14 residues). We showed that N‐glycosylation adjacent to the C‐terminal furin site regulates cleavage (Ashworth *et al*. 1999a), whilst mutations that perturb processing cause MFS, confirming their critical importance (Milewicz *et al*. 1995; Jensen *et al*. 2014). It seems likely that processing occurs at the cell surface as the first step in assembly, in association with HS proteoglycans which can activate furin convertases (Mayer *et al*. 2008) (Figure 5). We showed, using a proteomic approach, that at least some C‐terminal propeptide is retained within tissue microfibrils (Cain *et al*. 2006). The cleaved C‐terminal fragment (asprosin) was also recently identified as a glucogenic peptide hormone (Romere *et al*. 2016) (see also ‘Tall fibrillinopathies’).

## Homotypic fibrillin‐1 interactions

Studies of recombinant fibrillin‐1 and antibody epitope mapping (Reinhardt *et al*. 1996; Baldock *et al*. 2001) had suggested that N‐ to C‐terminal interactions after furin processing drive linear assembly (Figures 4a and 5) (see ‘Molecular dimensions and microfibril periodicity’). We and others showed, using recombinant fragments, that ‘furin‐processed’ N‐ and C‐terminal sequences interact strongly (Ashworth *et al*. 1999b; Trask *et al*. 1999; Lin *et al*. 2002; Marson *et al*. 2005). We also found interactions between N‐terminal fragments, and between processed C‐terminal fragments, which could support lateral association, as well as an interaction between the C‐terminal furin‐cleaved fragment and the processed C‐terminal region encoded by TB7 and cbEGFs 37–43 that contains the N‐terminal interaction site (Marson *et al*. 2005). Subsequently, it was found that secretion of fibrillin‐1 requires cbEGFs 41–43 to be present, suggesting that the C‐terminal postfurin site fragment fold backs to bind these cbEGFs and prevent N‐terminal interactions until secretion (Jensen *et al*. 2014). These data suggest how mutations within the postcleavage sequence can cause MFS. Interestingly, our proteomic study had revealed that some cleaved fragment is retained within zonular microfibrils (Cain *et al*. 2006). A further discovery was that, in the absence of the postcleavage sequence, the C‐terminal half of fibrillin‐1 forms beads that resemble those seen within microfibrils (Hubmacher *et al*. 2008). This multimerization also increased C‐terminal affinity for N‐terminal fibrillin‐1, and may be critical for linear and lateral assemblies.

## Interactions with HS

It is well known that supplementation of cultures with heparin, an analogue of HS, blocks microfibril deposition (Ritty *et al*. 2003; Tiedemann *et al*. 2001; Massam‐Wu *et al*. 2010), implying that HS plays a key regulatory role. Indeed, fibrillin‐1 interacts more than most other molecules of the extracellular matrix with heparin/HS (Tiedemann *et al*. 2001; Cain *et al*. 2005, 2008). Heparin interacts at both fibrillin‐1 furin cleavage sites, although we found using recombinant full‐length fibrillin‐1 that it does not block C‐terminal cleavage (unpublished data). Strong interactions between unprocessed fibrillin‐1 and heparin suggest that HS‐rich sites on cell surfaces ‘nucleate’ newly secreted fibrillin‐1 molecules prior to processing (Cain *et al*. 2008; Yadin *et al*. 2013). HS also binds C‐terminal sites (Cain *et al*. 2008) within cbEGFs 38–43; cbEGFs 41–43 are known to bind the N‐termini (Hubmacher *et al*. 2008) (see ‘Homotypic fibrillin‐1 interactions’); stronger binding was seen to multimerized C‐terminal fibrillin‐1 (Sabatier *et al*. 2014). It is unclear whether or not heparin/HS blocks N‐ to C‐terminal interactions (Cain *et al*. 2005; Sabatier *et al*. 2014); perhaps processing releases fibrillin‐1 from HS‐rich cell surfaces as a prerequisite for linear assembly. All of these HS‐driven molecular associations are likely to be directed by cell surface HS proteoglycans.

## Interactions with cells and conformation dependence

Fibrillin‐1 thus assembles pericellularly through terminal interactions, regulated by furin processing, and its RGD motif is not required (*see below*). What then is the role of direct cell receptor interactions with fibrillin‐1?

## Binding cell surface receptors

The fibrillin‐1 RGD motif within the interbead (see ‘Exposed fibrillin‐1 sites’) supports the adhesion of fibrillin‐1 molecules (Pfaff *et al*. 1996; Sakamoto *et al*. 1996; Bax *et al*. 2003, 2007; Lee *et al*. 2004; Jovanovic *et al*. 2007; McGowan *et al*. 2008; Cain *et al*. 2012) and assembled microfibrils (Kielty *et al*. 1992) to integrin receptors on cells. Fibrillin‐2 also supports RGD‐dependent cell adhesion (Brinkmann *et al*. 2010). These studies utilized recombinant fibrillin molecules or purified microfibrils in cell‐based adhesion assays.

**Figure 6:**
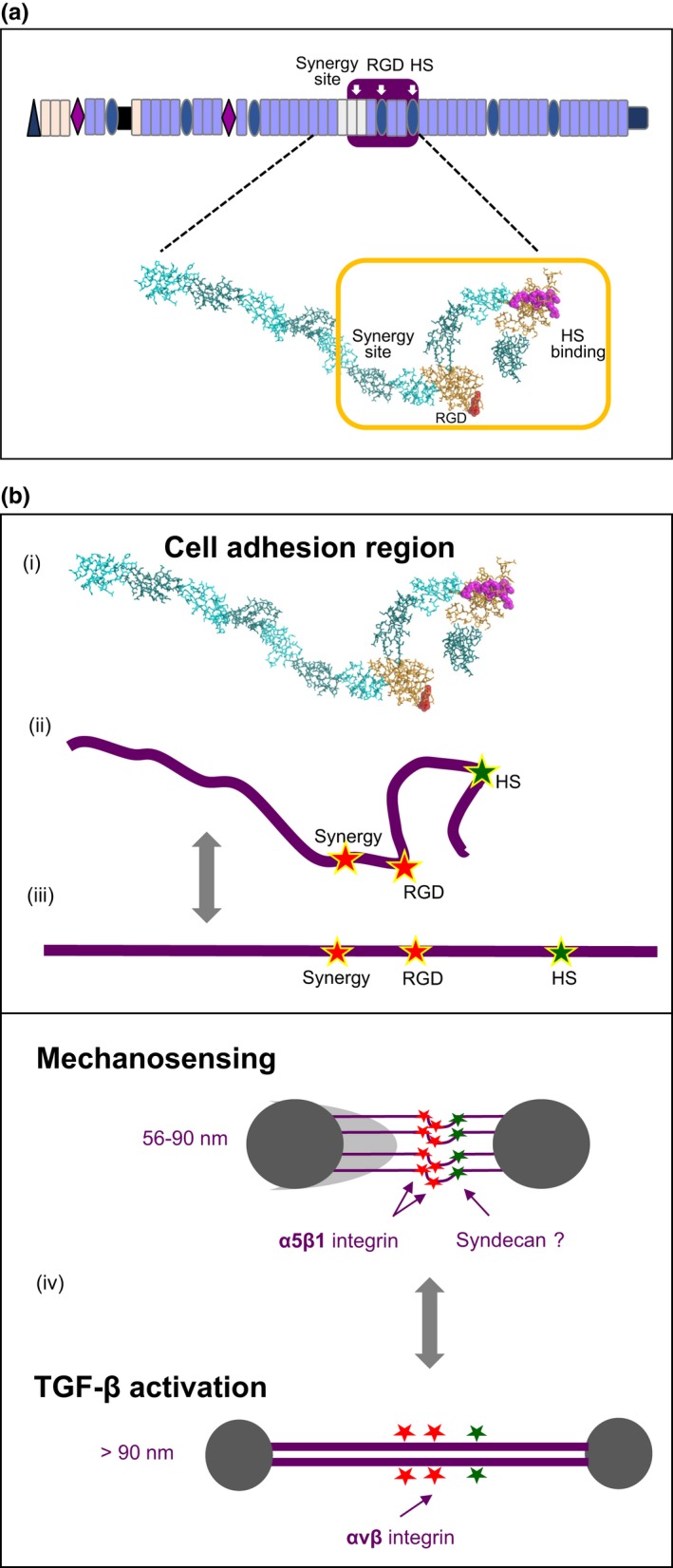
(a) Cell adhesion region of fibrillin‐1. The diagram shows TB4 containing the RGD motif and an upstream synergy site important for binding conformation‐sensitive α5β1 integrin (Bax *et al*. 2007), and TB5 containing an HS‐binding site (Cain *et al*. 2009). The modelled multidomain organization (from Cain *et al*. 2009; image reproduced from PLoS One, under the Creative Commons Attribution Licence) shows untensioned fibrillin‐1 fragment PF17‐1 (cbEGFs 16–22; TB4; cbEGFs 23,24; TB5; cbEGF 25). It highlights the likely orientation of interbead RGD and synergy sites, and HS‐binding domain in untensioned state (Cain *et al*. 2008, 2012). It is derived from solution small‐angle X‐ray scattering analysis in the presence of calcium. The model also shows (in purple) the position of an in‐frame deletion in TB5 which causes WMS (Faivre *et al*. 2003). (b) Microfibril tensometer model. The hypothetical model builds on the solution structure of the untensioned fibrillin‐1 fragment PF17‐1 in the presence of calcium (as in Figure 6a). Tensional forces will extend the cell adhesion region, disrupting conformation‐sensitive binding to integrin α5β1 at focal adhesions. These structural changes will be directly sensed by cells, and may support a switch to engagement of αvβ3/5 integrins which would trigger TGF‐β activation as a repair response. [Colour figure can be viewed at wileyonlinelibrary.com].

We showed using multidomain fibrillin‐1 fragments expressed by mammalian cells to ensure correct disulphide bonding and N‐glycosylation, that the fibrillin‐1 RGD region binds α5β1 in a highly conformationally dependent interaction, as well as αvβ3/β5 integrins (Bax *et al*. 2003). We also identified a high‐affinity HS‐binding site in TB5, which is downstream from the RGD‐containing TB4; together with an α5β1 integrin synergy site upstream from TB4, these regions comprise the fibrillin‐1 cell adhesion region (Figure 6a) (Bax *et al*. 2007).

The fibrillin‐1 cell adhesion region thus has striking similarities to that of fibronectin, despite no primary sequence correlation. Yet, although fibronectin assembly is directed by integrin‐mediated cell surface forces (Schwarzbauer & DeSimone 2011), integrin adhesion to fibrillin‐1 is not needed to assemble individual microfibrils *in vitro* (Hubmacher *et al*. 2014b); moreover, mutations in the RGD‐containing TB4 which cause SSS, and a corresponding targeted RGD‐to‐RGE mutation in mice, both induce abundant (albeit disorganized) microfibrils (see ‘Clues from the “short” fibrillinopathies’). Increased microfibrils could be a consequence of enhanced TGF‐β signalling because of altered integrin‐mediated adhesion. Moreover, fibrillin microfibrils precede the appearance of the RGD cell adhesion motif by over 500 million years (Piha‐Gossack *et al*. 2012; see also ‘Evolution of the fibrillin superfamily’).

The solution structure of the fibrillin‐1 cell adhesion region was determined by small‐angle X‐ray scattering using calcium‐bound multidomain fragments (Cain *et al*. 2012). It is clearly not linear in untensioned state, thereby altering the distance between RGD motif and synergy site, and the HS‐binding site in TB5, compared to extended state (Figure 6a). Fibronectin studies had shown that the distance between its RGD and synergy site (32Å) is crucial for its interactions with α5β1 integrin and that extending this distance to 55Å by mechanical forces can turn off binding to α5β1 (Krammer *et al*. 2002). The same outcome probably occurs in extended microfibrils (see ‘Microfibrils as structural tensometers’).

## Microfibrils as structural tensometers?

Cook *et al*. (2014) first proposed that microfibrils may contribute to mechanosignaling. Combined microfibril data, summarized in this review, are consistent with the conceptual hypothesis proposed here that fibrillin‐1 microfibrils are hypersensitive tensometers (tensional gauges) that enable cells to sense, and respond to changes in the mechanical status of tissues (Figure 6b). They may achieve this by extending within their reversible range (~56–90 nm), as tissues stretch, with ‘straightening’ of the interbead where the cell adhesion site is located (Figure 4b). These structural changes would disrupt the highly conformation‐sensitive binding sites needed for α5β1 integrin (RGD with upstream synergy region; TB4) and HS (probably syndecan‐4; TB5); both receptors are the essential focal adhesion components and cellular mechanosensors (Couchman *et al*. 2015; Sun *et al*. 2016). Loss of adhesion to α5β1, and corresponding gain of adhesion to αvβ integrins (which do not have such conformational constraints but can activate latent TGF‐β from matrix; see LTBPs) would profoundly alter cell signalling and trigger responses such as TGF‐β activation to repair matrix. The inherent property of microfibrils to extend and retract in normal dynamic tissues may sustain α5β1 integrin interactions, and focal adhesion kinase activity. However, pathological extension could induce conformation‐sensitive ‘flipping’ of cell adhesion from α5β1 to αvβ integrins. In this way, the tensometer model reconciles the elastomeric essence of microfibrils with their ability to provoke robust TGF‐β responses. This model is also consistent with the finding by Cook *et al*. (2014) that reduced focal adhesion kinase signalling (downstream of the focal adhesion receptor integrin α5β1) is a consequence of fibrillin‐1 deficiency.

## Deposition of microfibril bundles

The formation of microfibril bundles is poorly understood. Early microscopy of developing aorta indicated that it occurs in association with dense (focal adhesion) plaques on subendothelial cells, with forming bundles extending into the matrix (Davis 1994). It suggests that microfibrils may be ‘bundled’ by cellular interactions at HS‐rich adhesions.

## Fibronectin enhances microfibril deposition by mesenchymal cells

We and others showed that the cell adhesion molecule fibronectin is needed for the robust deposition of microfibril bundles by cells of mesenchymal origin, such as fibroblasts and smooth muscle cells (Kinsey *et al*. 2008; Sabatier *et al*. 2009; Zilberberg *et al*. 2012). Knockdown of fibronectin, or genetic mutation of its RGD motif, ablated microfibril networks in culture models. Microfibril deposition was restored by adding fibronectin.

Our later study showed that, unlike mesenchymal cells, certain epithelial cells (retinal epithelial cells and podocytes) were not dependent upon fibronectin for microfibril deposition although they did require α5/α8β1 integrin and syndecan‐4 (Baldwin *et al*. 2014). Moreover, the epithelial–mesenchymal status of the cells regulated whether or not fibronectin was needed for microfibril deposition. In this context, ‘mechanically driven cross‐talk’ between integrins and epithelial cadherins is known to control the balance of matrix assembly and tissue formation by these cellular receptors (Mui *et al*. 2016).

It was also shown that fibronectin‐null embryonic cells can assemble microfibrils (Dallas *et al*. 2005), whilst invertebrate microfibrils assemble in abundance despite having no fibronectin gene. Moreover, although fibronectin networks based on gelatin‐binding domain interactions were necessary for microfibrils, fibronectin–fibrillin interactions were not (Sabatier *et al*. 2013). Fibrillin‐1 also does not bind strongly to fibronectin (Hubmacher *et al*. 2008; Cain *et al*. 2009; Sabatier *et al*. 2009), although the interaction is strengthened by fibrillin‐1 multimerization (as in a microfibril) (Sabatier *et al*. 2014).

Why then might fibronectin be required for mesenchymal cells to deposit microfibrils? We proposed that, in mesenchymal cultures and tissues with few HS‐rich cell–cell junctions, fibronectin is needed as a highly effective inducer of focal adhesions which are HS‐rich clusters of syndecan‐4 with α5β1 integrin receptors (Baldwin *et al*. 2014; Couchman *et al*. 2015). Such a role for fibronectin would fit its evolutionary appearance alongside the need for robust tissues, such as elastic arteries for high‐pressure circulatory systems.

## How might HS‐rich cell surface zones influence microfibril deposition?

Data indicate that HS‐rich surface areas strongly influence microfibril bundle formation, and may also enable cells to respond to altered matrix mechanics.

### Clues from the ‘short’ fibrillinopathies

Mutations in the fibrillin‐1 cell adhesion region, both RGD‐containing TB4 (SSS) and HS‐binding TB5 (WMS, AD, GD) domains, result in abundant but disorganized microfibrils and generalized thickening or fibrosis of skin. Thus, defective cell adhesion does not block assembly but instead disrupts microfibril deposition and increases matrix abundance, implying altered cell signalling responses.

Mice with a fibrillin‐1 RGD‐to‐RGE mutation recapitulated SSS phenotype, implicating the fibrillin‐1 RGD directly in this phenotype. Their fibrotic skin phenotype was prevented by treatment with integrin β1‐activating or β3‐blocking antibodies, and largely reversed by antagonizing TGF‐β (Gerber *et al*. 2013). The β3 integrin effect might be explained by binding to latency‐associated propeptide RGD, thereby releasing TGF‐β from matrix stores of large latent complexes. Activating β1 integrins will include α5β1 integrin, an essential component of focal adhesions that mechanosense matrix (Bax *et al*. 2003; Schwarzbauer & DeSimone 2011).

All but one of the dominant fibrillin‐1 mutations that cause WMS or the acromelic dysplasias are in HS‐binding TB5, and the molecules are secreted (Le Goff *et al*. 2011; Jensen *et al*. 2015). We defined the HS‐binding site on fibrillin‐1 TB5 by comparing fibrillin‐1 and fibrillin‐2 TB5 domains, then showed that WMS, AD and GD mutations map to this HS‐binding site in TB5 (Cain *et al*. 2012). Moreover, all disease‐causing mutations tested were shown to inhibit HS binding. Thus, HS interactions are directly implicated in the ‘short’ fibrillinopathies, perhaps altering ordered microfibril ‘bundling’ and/or tensional cell signalling.

### Clues from ADAMTS10 and HS‐rich cell interfaces

Autosomal recessive mutations in ADAMTS10 cause a WMS phenotype that is indistinguishable from that is caused by fibrillin‐1 mutations (Le Goff & Cormier‐Daire 2012). ADAMTS10 is a member of the ‘a disintegrin and metalloproteinase with thrombospondin motifs’ family. It is unusual in having a degenerate furin cleavage site that is not processed unless experimentally mutated, and thus is effectively inactive (Kutz *et al*. 2011; Cain *et al*. 2016). Supplementation of cultured bovine nuchal ligament fibroblasts with medium containing recombinantly expressed ADAMTS10 accelerated deposition of microfibrils; in comparison, fibroblasts from a WMS patient deposited few microfibrils (Kutz *et al*. 2011). ADAMTS10 was found to bind fibrillin‐1, and it was proposed that ADAMTS10 enhances microfibril deposition through direct interactions. Although not associated with a fibrillinopathy to date, ADAMTSL6 was also shown in a genetic mouse model to bind fibrillin‐1 and enhance microfibril deposition (Tsutsui *et al*. 2010). ADAMTSL6 is related to ADAMTS10 but lacks a catalytic domain and thus, like ADAMTS10, is inactive.

Another explanation for how ADAMTS10 stimulates microfibril deposition was revealed in our recent study (Cain *et al*. 2016). Purified recombinant ADAMTS10 and its closest homologue, ADAMTS6, both bound fibrillin‐1, heparin and syndecan‐4 ectodomain. However, only ADAMTS6 was processed, active and disrupted syndecan‐4, which is an essential component not only of focal adhesions but also epithelial HS‐rich cell–cell junctions, both key sites of tension sensing (Gopal *et al*. 2016). ADAMTS10 strikingly enhanced focal adhesions and epithelial cell junctions, and microfibril deposition, whereas ADAMTS6 had catastrophic effects on HS‐rich interfaces and on microfibrils.

**Figure 7:**
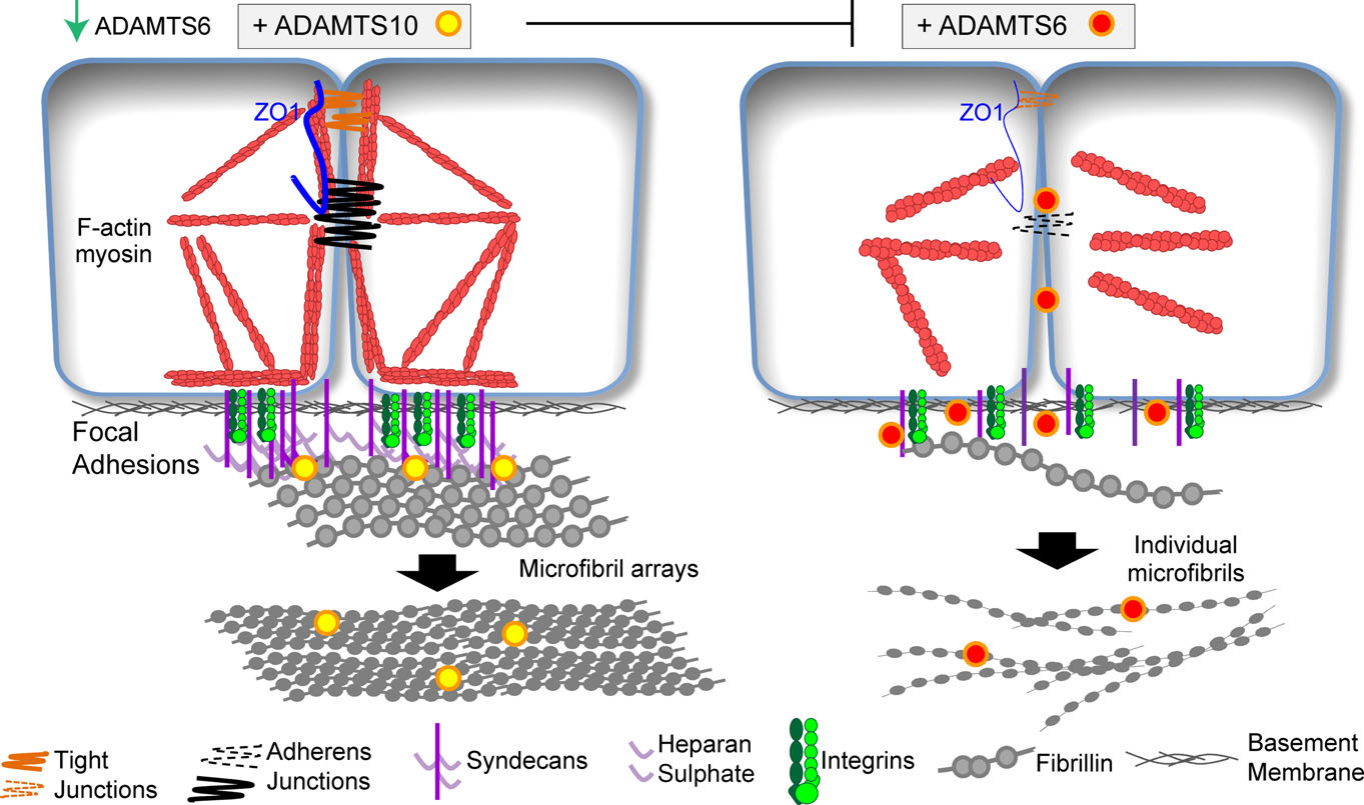
Model of how ADAMTS10 can support HS‐rich epithelial cell junctions. We have proposed, on the basis of experimental evidence (Cain *et al*. 2016), that inactive ADAMTS10 enhances cell surface syndecan‐4 and HS, cell–cell junctions and focal adhesions, but that its active homologue ADAMTS6 degrades syndecan‐4 and disrupts cell–cell junctions and focal adhesions. Such HS‐rich sites are likely to be critical for microfibril assembly. This image is reproduced with permission from Science Reports, under the Creative Commons Attribution Licence. [Colour figure can be viewed at wileyonlinelibrary.com].

Notably, in these epithelial cultures, ADAMTS10 was expressed at extremely low levels (Cain *et al*. 2016). It was also not detected in a proteomic analysis of zonules, despite the fact that ectopia lentis is often a feature of WMS (De Maria *et al*. 2017), although it did localize to ciliary zonules as shown by immunogold labelling (Kutz *et al*. 2011). Thus, it seems improbable that ADAMTS10 is widely available at the abundant levels needed to interact directly with fibrillin‐1 to support assembly. Moreover, ADAMTS10 only appeared in early vertebrate evolution (Nicholson *et al*. 2005), so invertebrate microfibrils do not need it to assemble. Despite low levels, we found that ADAMTS10 expression did significantly suppress levels of ‘damaging’ ADAMTS6. In summary, ADAMTS10 may stimulate microfibril deposition by enhancing HS‐rich adhesions and epithelial cell junctions; it may achieve the latter in part by controlling ADAMTS6 expression and activity (Figure 7).

It is striking that members of the related ADAMTSL family, which lack the ADAMTS catalytic domain and are thus inactive, cause similar fibrillinopathies, perhaps by comparable mechanisms. ADAMTSL2 mutations cause GD (Le Goff *et al*. 2008) and Musladin–Leuke syndrome in beagle dogs (Bader *et al*. 2010), whilst ADAMTSL4 deficiency causes autosomal recessive isolated ectopia lentis (IEL) and ectopia lentis et pupillae (Collin *et al*. 2015). There is also evidence for epithelial involvement in these diseases, reminiscent of the microfibril defects seen at the dermal–epidermal junction in ‘short fibrillinopathies’ caused by fibrillin‐1 mutations. Homozygous ADAMTSL2 null mice have severe bronchial epithelial dysplasia (Hubmacher *et al*. 2015), whilst ADAMTLS4 mice with a non‐sense mutation have de‐differentiated retinal pigment epithelium defects (Collin *et al*. 2015).

## Summary and future directions

As outlined above, recent years have seen huge progress in defining microfibril biology, especially their construction and how mutations affect structure and function. Much emphasis has been placed on the growth factor regulatory features of microfibrils, which – although important – have deflected attention from microfibrils as indispensible structural macromolecules of matrix. This review has put forward the hypothesis that microfibrils are in fact structural tensometers, a model for future testing that directly integrates their structural and growth factor regulatory roles.

Many questions still remain about how microfibrils form and support tissue homeostasis, and how newly revealed features such as phosphorylation impact function. Improved therapies for fibrillinopathies and degeneration of ageing elastic tissues await insights into these issues.

## Funding source

Past research funding from the Medical Research Council, Biotechnology and Biological Sciences Research Council, Wellcome Trust, British Heart Foundation, Arthritis Research UK and Royal Society is gratefully acknowledged. The Wellcome Trust Centre for Cell‐Matrix Research, University of Manchester, is supported by core funding from the Wellcome Trust [grant number 203128/Z/16/Z]. Fibrillin research from the Kielty laboratory over 25 years was supported by a talented research team reflected in our publications from 1991 to 2016. The author is indebted to C. A. Shuttleworth and C. Baldock for excellent collaborations over many years.
